# Integrated Analysis of Testicular Histology, Sperm Quality, and Gene Expression (*TGFB2*, *DMRT1*) in Rooster Semen (*Gallus gallus domesticus*)

**DOI:** 10.3390/ani16020225

**Published:** 2026-01-12

**Authors:** Anastasiya Ivershina, Yuliya Silyukova, Elena Fedorova, Olga Stanishevskaya, Irina Mirzakaeva, Marina Pozovnikova

**Affiliations:** Russian Research Institute of Farm Animal Genetics and Breeding—Branch of the L.K. Ernst Federal Research Center for Animal Husbandry (RRIFAGB), Tyarlevo, Moskovskoe Shosse, 55a, 196625 St. Petersburg, Russia; nastya_ivershina@mail.ru (A.I.);

**Keywords:** roosters, histology, sperm, testes, spermatogenesis, morphometry, correlation analysis, *DMRT1*, *TGFB2*, gene expression

## Abstract

The study of the relationship between testicular morphology and sperm quality is a pressing issue, for which molecular genetic approaches, including quantitative analysis of gene expression, are being implemented. The aim of this study was to identify correlations between the histomorphological structure of the testes, fresh sperm parameters, and the expression level of key spermatogenesis genes—*TGFB2* and *DMRT1*—in roosters. The experiment was conducted on 10 Russian Snow White roosters aged 28–32 weeks. Sperm quality was assessed by volume, sperm concentration, total and progressive motility, and viability; histological analysis of the rooster testes was performed. The relative expression of the *TGFB2* and *DMRT1* genes in sperm was analyzed. Multiple correlation analysis of the data was conducted. A positive correlation was found between ejaculate volume and the number of spermatogonia (*p* = +0.651), a negative correlation between ejaculate volume and the number of second-order spermatocytes (*p* = −0.704), a negative correlation between the total cross-sectional area of the seminiferous tubules of the testes and sperm viability (*p* = −0.782), a negative correlation between the number of seminiferous tubules and the average diameter of their cross-section (*p* = −0.685), and a positive correlation between total and progressive sperm motility (*p* = +0.794). Analysis of *TGFB2* and *DMRT1* gene expression in sperm demonstrated a certain relationship between molecular genetic mechanisms and histomorphometric parameters. The expression level of the *DMRT1* gene, which plays a key role in sex determination in birds during embryogenesis, had a number of negative correlations with such parameters as testicle weight (r = −0.782), total/progressive sperm motility (r = −0.552; r = −0.612), and viability (r = −0.552). Expression of the *TGFB2* gene had no significant relationship with the studied parameters, but correlation analysis revealed a moderate positive relationship (r = +0.321) with *DMRT1* gene expression. The data obtained indicate the expediency of integrating morphometric, cellular, and molecular analysis for an objective assessment of rooster reproductive function.

## Simple Summary

The aim of this study was to identify correlations between testicular histomorphology, fresh semen parameters, and the expression level of key spermatogenesis genes—*TGFB2* and *DMRT1*—in roosters. Analysis of *TGFB2* and *DMRT1* gene expression in fresh semen demonstrated a close relationship between molecular genetic mechanisms and histomorphometric parameters. Thus, the expression level of the *DMRT1* gene, which is key in determining sex in birds during embryogenesis, showed a number of negative correlations with parameters such as testicular weight, total/progressive sperm motility, and viability. *TGFB2* gene expression in fresh semen showed no significant association with the studied parameters, but correlation analysis revealed a moderate positive association with *DMRT1* gene expression. The results of this study support the need for an integrated approach to assessing the reproductive performance of males and the quality of sperm produced.

## 1. Introduction

The reproductive system of male poultry, in particular, *Gallus gallus domesticus*, is a determining factor in their breeding worth, since it is on its structural and functional integrity that the success of spermatogenesis, the quality of the resulting sperm production and the fertility of the semen depend [1,2]. Unlike mammals, the testicles of birds are paired internal organs located in the abdominal cavity, which causes them to function at high temperatures—about 41–42 °C [3,4,5]. Spermatogenesis occurs in the seminiferous tubules, where all its stages are consistently realized: from the proliferation of primary germ cells, spermatogonia, to the formation of mature germ cells, spermatozoa [6]. Their full-fledged development is directly related to the quality of the semen obtained, which is the most important condition determining the effectiveness of artificial insemination in breeding programs [7]. According to scientific studies devoted to the assessment of fresh and cryopreserved semen in birds, it was found that indicators such as ejaculate volume, sperm concentration, motility and viability directly affect semen fertility, which, as a result, affects egg fertilization [7,8].

The seminiferous tubules (*tubulus seminiferous*) are a structural and functional unit of the testicle, which is a convoluted tubular structure lined with spermatogenic epithelium [9]. The seminiferous tubules are surrounded by a basement membrane and peritubular myoid cells, which provide the contractile function necessary for the movement of spermatozoa to the outflow ducts of the tubule [10]. The spermatogenic epithelium is formed by several cell populations at various stages of differentiation: spermatogonia, spermatocytes of the first and second orders, and spermatids, from which mature spermatozoa are formed [11,12]. Morphometric parameters of the seminiferous tubules, such as diameter, cross-sectional area, height of the spermatogenic epithelium and the inner diameter (lumen) of the tubule, are quantitative indicators characterizing the intensity and effectiveness of spermatogenesis [9,13,14]. Studies conducted on chickens have shown significant variability of these parameters depending on the age, breed, physiological state and direction of poultry productivity [9,15,16]. For example, in a study by Chinese scientists, it was found that in broiler chickens at the age of 1 week, the diameter of the seminal tubules was 40–50 µm and increased to 90–120 µm by 4 months, which corresponds to the age of puberty of roosters and the beginning of active spermatogenesis. In adult roosters, this indicator varies from 150 to 305 µm [15]. In the active reproductive phase, as a rule, the presence of all stages of spermatogenic cells with a predominance of spermatids is observed in the seminiferous tubules, which indicates the normal state of the male’s reproductive system [17,18]. In addition, Sertoli cells are an essential structural component of the spermatogenic epithelium. These cells are involved in the formation of the hematotesticular barrier and support developing gametes, so their nuclei can extend from the basement membrane to the lumen of the seminal tubule [19]. It is known that the concentration of Sertoli cells has a strong positive correlation with the testicles’ weight in roosters, which also confirms the fundamental importance of these cells in spermatogenesis in birds [20].

The search for links between the morphological structures of the testicles and the quality of the semen is the subject of many scientific studies. For example, it has been found that in mammals, testicle weight positively correlates with ejaculate volume and the total number of spermatozoa, and indicators such as diameter and cross-sectional area of the seminiferous tubules are associated with sperm concentration and motility [21]. It is also known that various disorders of the histological structure, such as a decrease in the height of the spermatogenic epithelium, an increase in the diameter of the tubule lumen, and germ cell apoptosis, are accompanied by a decrease in semen quality, and, as a result, a deterioration in its fertility [22]. In addition to traditional methods of assessing fertility in sires, for example, through spermograms, as well as microscopic and histological analysis of the gonads, molecular genetic approaches have been increasingly introduced in recent years. These include quantitative analysis of gene expression using real-time PCR (qPCR), genome-wide sequencing, analysis of polymorphisms in regulatory genes, and the use of CRISPR/Cas technology for the functional study of the role of individual genes in reproduction [23]. In our study, we focus on two genes, *DMRT1* and *TGFB2*, which are key regulators of spermatogenesis and the development of male reproductive organs in vertebrates, including birds. The *DMRT1* gene (doublesex- and mab-3-related transcription factor 1) is a conserved transcription factor localized on the Z chromosome in birds, which is considered critically important for determining the male sex and maintaining testicle function at all stages of ontogenesis [24]. In chickens, *DMRT1* acts dose-dependently: individuals with the ZZ genotype (males) have a sufficient level of its expression for testicle development, whereas if the gene expression is disrupted, their partial or complete feminization is possible [25,26]. At the molecular level, the *DMRT1* gene regulates Sertoli cell differentiation, supports proliferation, and prevents premature entry of spermatogonia into meiosis, ensuring constant replenishment of the stem cell population [27,28]. Violation of its expression leads to a decrease in sperm quality and, in extreme cases, to complete infertility of the male [29]. Another gene, *TGFB2* (transforming growth factor beta 2), which belongs to the family of TGF-β signaling molecules, also plays an important role in the regulation of spermatogenesis and [30]. Mammalian studies have shown that TGF-β signaling is involved in the maintenance of spermatogonial stem cells, regulates their differentiation and apoptosis, thus influencing the dynamics of spermatogenesis [30,31]. Also, in one study in mice, correlations were found between the expression levels of TGF-β family genes and sperm quality, including sperm motility and viability [32]. Studies of complete transcriptomics of the testicle and appendage in roosters have revealed that the TGF-β pathway is one of the key signaling pathways regulating spermatogenesis, which interacts with pathways such as MAPK and mTOR [33]. This indicates that *TGFB2* is integrated into complex regulatory networks that control sperm production in males. In addition, it was found that the *TGFB2* gene is expressed in the seminal fluid of roosters, where it presumably plays an immunomodulatory role [34].

Our study is devoted to the study of multilevel mechanisms of regulation of reproductive function in roosters *(Gallus gallus domesticus)*. Understanding the relationships between the morphology of the testicles, the expression level of key regulatory genes such as *DMRT1* and *TGFB2*, as well as the functional parameters of rooster sperm, may open up new opportunities for improving reproductive performance in poultry farming by using molecular genetic methods in breeding and developing new biotechnological approaches.

## 2. Materials and Methods

For the study, roosters of the Russian Snow White breed (n = 10) aged 28–32 weeks of life were selected from the bioresource collection “Genetic Collection of Rare and Endangered Chicken breeds” (Russian Research Institute of Farm Animal Genetics and Breeding—Branch of the L.K. Ernst Federal Research Center for Animal Husbandry (RRIFAGB), St. Petersburg, Russia) according to the reaction to abdominal massage and qualitative indicators of fresh semen—ejaculate volume (mL), sperm concentration (billion/mL), total and progressive motility (%), viability (%). The chickens were kept in individual cages with the same feeding, watering and light conditions, which correspond to the direction of productivity of the breed.

### 2.1. Collection and Processing of the Data

Starting from the age of puberty, the roosters were accustomed to abdominal massage and were used in the semen extraction mode 2 times a week. The semen was selected in accordance with GOST 27267-2017 [35]. Ejaculate volume (mL) was measured with a graduated pipette, and sperm concentration (billion/mL) was measured using an Accuread Photometer (photometer Accuread® IMV Technologies, L’Aigle, France). Total and progressive motility (TM and PM, %) were determined using the CASA ArgusSoft-Poultry imaging system (Motic BA410E, Motic, Xiamen, China; ArgusSoft software-1, St. Petersburg, Russia) at magnification ×100. The viability of the frozen/thawed semen was evaluated by staining with eosin/nigrosin (EN) dye, visualized on a Motic BA410E phase contrast microscope (Motic BA410E, Motic, Xiamen, China) at ×1000 magnification under immersion oil. At least 200 cells were evaluated in each sample. The pink-colored cells were considered damaged (dead). A two-component medium was used to dilute the semen: 1.8 g of glucose, 2.8 g of monosodium glutamate per 100 mL of distilled water (RRIFAGB, patent No. 2485816 of the Russian Federation in 2013). The semen quality was assessed in 4 repetitions for each individual.

### 2.2. Preparation and Analysis of Histological Sections

Euthanasia of roosters was performed by cervical dislocation. The abdominal cavity was opened, and the right testicle was extracted. The testicles were weighed on digital scales, fixed in a 10% solution of neutral formalin (pH 7.2–7.4), purified with xylene, and then treated with paraffin. Serial sections with a thickness of ~4 µm were prepared from waxed samples on an RMD-3000 rotary microtome (MedTechnicaPoint Techology, St. Petersburg, Russia), which were stained with hematoxylin/eosin and analyzed under a microscope BIOSCOPE-4 (LOMO PLC, St. Petersburg, Russia) (magnification ×100, ×200, ×400) with an imaging system and photo/video software for the fixations. Morphohistological analysis of the composition of the spermatogenic epithelium was determined on 10 randomly selected cross-sections on selected fields measuring 100 × 100 µm^2^, and the main types of cells of the spermatogenic epithelium were calculated. The average values (M) and standard errors (±SE) were calculated for all indicators. The histological analysis included the calculation and evaluation of the following indicators: number of seminiferous tubules in the field of view (magnification ×100); average diameter of the cross sections of the seminiferous tubules (µm) (magnification ×200); total cross-sectional area of the seminiferous tubules in the field of view (S, µm^2^); number of cells of the spermatogenic epithelium: spermatogonia, spermatocytes of the first and second order, spermatids and Sertoli cells (increase ×400).

### 2.3. Isolation of RNA

Aliquots with a volume of 100–150 µL were selected from each individual semen sample for further RNA isolation. Total RNA samples were isolated from germ cells (sperm).

RNA isolation from rooster sperm was performed according to the following protocol. Total RNA isolation from rooster sperm was performed using the commercial Lira-Karib kit (Biolabmix LLC, Novosibirsk, Russia) according to the manufacturer’s recommendations, with individual modificationns.

#### 2.3.1. Preliminary Purification of Sperm from Somatic Cells

The ejaculate (100–200 µL) was washed twice in 1–1.5 mL of saline (0.9% NaCl) at 800× *g* for 5 min at 4 °C. The supernatant containing sperm was carefully transferred to a new tube, avoiding disturbing the sediment enriched with somatic cells. After the second wash, the sperm were pelleted by centrifugation at 600× *g* for 5 min at 4 °C. The supernatant was removed, and the sediment was used for lysis.

#### 2.3.2. Cell Lysis and RNA Extraction

A total of 1 mL of Lira-Carib reagent was added to the sperm sediment (per 10–50 μL of original semen). 20 μL of β-mercaptoethanol (final concentration ~0.2%) was added to ensure effective RNase inhibition. The samples were thoroughly homogenized by vortexing for 30–60 s until the sediment was completely dissolved (using ceramic beads). The samples were incubated at room temperature for 5 min to completely dissociate the nucleoprotein complexes.

#### 2.3.3. RNA Precipitation

In total, 200 µL of chloroform per 1 mL of Lira-Carib reagent was added to the lysate. The tubes were vigorously shaken for 15–30 s, then incubated at room temperature for 2–3 min. The tubes were centrifuged at 12,000× *g* for 15 min at 4 °C. The upper (aqueous) phase, containing the RNA, was carefully transferred to a new RNase-free tube, avoiding the interphase layer and the organic phase. Then 0.5 mL of isopropanol was added to the aqueous phase per 1 mL of the original Lira-Carib reagent. The mixture was incubated for 10 min at room temperature. The mixture was centrifuged at 12,000× *g* for 10 min at 4 °C. The supernatant was carefully removed, leaving the white RNA sediment at the bottom. The sediment was washed with 1 mL of 75% ethanol (in RNase-free water). The sediment was vortexed or pipetted to resuspend. The sediment was centrifuged at 7500× *g* for 5 min at 4 °C. The supernatant was removed, and the sediment was allowed to dry for 1–2 min.

#### 2.3.4. RNA Dissolution

Purified RNA was dissolved in 20–50 µL of RNase-free water or TE buffer (10 mM Tris-HCl, 1 mM EDTA, pH 7.0–8.0). The mixture was incubated at 55–60 °C for 10 min to completely dissolve. The dissolved RNA was stored at −80 °C.

#### 2.3.5. RNA Quality and Quantity Control

RNA concentration and purity were assessed spectrophotometrically using a NanoDrop2000c instrument (Thermo Fisher Scientific, Philadelphia, PA, USA). Samples with an A260/A280 ratio in the range of 1.9–2.1 and an A260/A230 ratio > 2.0 were considered suitable for subsequent qPCR analysis. The average sample concentration was 400 μg/mL.

### 2.4. Analysis of Relative Gene Expression

The level of relative expression was determined by a two-step method, which included (1) cDNA synthesis with reagents from the M–MuLV-RH Reverse Transcriptase kit (Biolabmix LLC, Novosibirsk, Russia) and (2) PCR-RV using BioMaster RT-PCR SYBR Blue (2×) (Biolabmix LLC, Novosibirsk, Russia) on the QuantStudio™ 5 Real Time PCR System (Thermo Fisher Scientific, Philadelphia, PA, USA). RT-PCR reactions for each sample were performed in three repeats. The arithmetic mean was used for subsequent calculations. Relative quantification of gene expression was performed using the ΔΔCt method. As no validated housekeeping genes were available for chicken spermatozoa at the time of the experiment, we used *GAPDH* as a reference gene, with its expression measured in kidney tissue; this approach allowed for intragroup comparisons within sperm samples. The selection of the primer sequence for the *DMRT1*, *TGFB2*, and *GAPDH* genes was performed using the BLAST program (https://www.ncbi.nlm.nih.gov/, accessed on 20 November 2025). The primers were synthesized by IH-BFM SB RAS (Institute of Chemical Biology and Fundamental Medicine, Siberian Branch of the Russian Academy of Sciences, Novosibirsk, Russia) (Table 1).

### 2.5. Statistical Processing of Results

To identify possible links between the morphometric, cellular and functional parameters of the testicles, a correlation analysis was performed using the software Statistica 10. (StatSoft, Inc., Tulsa, OK, USA). The statistical significance of the indicators was calculated using Spearman’s coefficient (*p* < 0.05). The visualization of the correlation matrix was carried out in the form of a “heat map” graph created using the Python 3.12 (Python Software Foundation, Wilmington, DE, USA) scripting programming language. Statistical significance of Spearman’s rank correlation coefficients was assessed at α = 0.05. For a sample size of n = 10, the critical value of |r_s_| is 0.648; therefore, correlations with |r_s_| ≥ 0.648 were considered statistically significant (*p* < 0.05).

## 3. Results

### 3.1. Analysis of Rooster Semen

To evaluate the obtained semen, 4 samples of fresh ejaculate were taken from each rooster (n = 10). The average values of qualitative indicators are presented in Table 2.

The volume of ejaculate produced ranged from 0.20 mL to 0.79 mL, which corresponds to the norm for mature roosters of egg breeds. The sperm concentration ranged from 1.08 billion/mL to 3.26 billion/mL, which is also within the normal range. The indicators of total and progressive motility of spermatozoa in fresh semen had significant individual variability: TM ranged from 65% to 91%, and PM—from 57% to 79%. Both indicators had rather high values, which is typical for males in the phase of active puberty. The average value for viability was 76.5%, which indicates a high survival rate of germ cells and the suitability of sperm for artificial insemination (at least 70% according to GOST 27267-2017 [35]).

**Figure 1:**
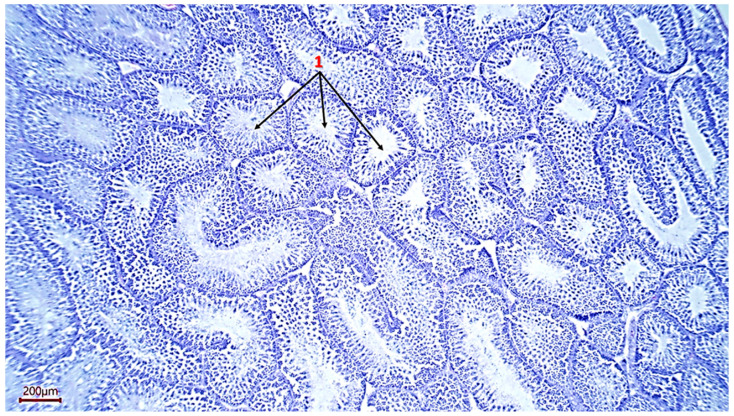
Histological section of the testicle. 1—seminiferous tubules of a rooster (magnification ×100, scale 200 µm).

**Figure 2:**
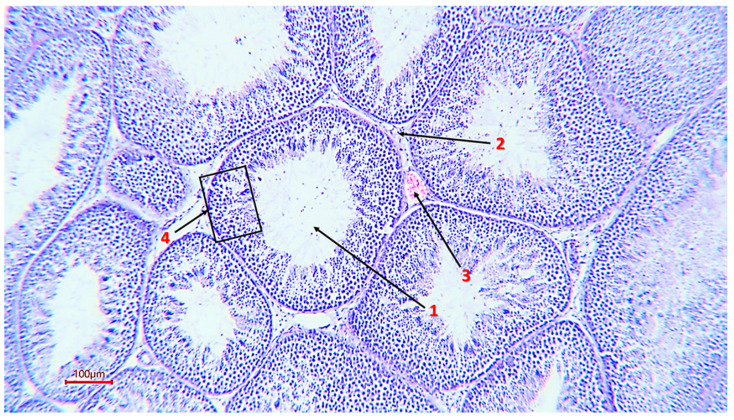
The general structure of the rooster’s seminiferous tubule (magnification ×200, scale 100 µm), 1—tubule lumen; 2—interstitial tissue; 3—capillary; 4—spermatogenic epithelium.

The weight of the roosters’ testicles ranged from 7.16 g to 25.47 g, which indicates individual characteristics in males, which are obviously related to hormonal, genetic or other factors (Table 3). Histological analysis of the testicle structure revealed a typical organization for birds—a multitude of densely packed seminiferous tubules lined with multilayer spermatogenic epithelium. Seminiferous tubules of small and large size, separated by interstitial tissue, are clearly visible on the analyzed samples. At the same time, almost all sections have seminiferous tubules having an elongated and sinuous shape, which indicates a complex anatomical arrangement of the seminal tubules in the testis (Figure 1 and Figure 2).

**Figure 3:**
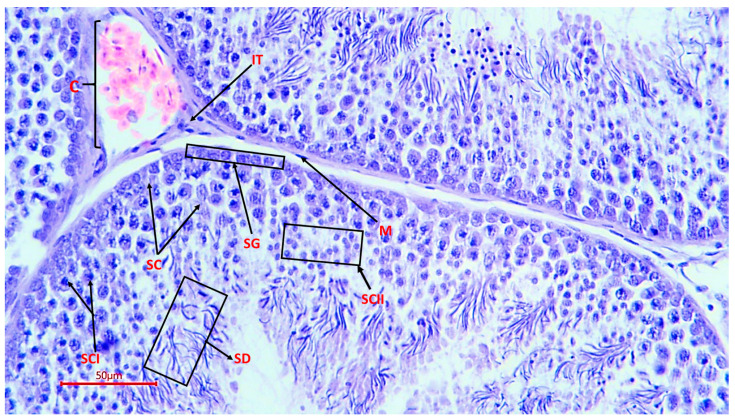
Spermatogenic epithelium of the seminiferous tubule of a rooster (magnification ×400, scale 50 µm), SG—spermatogonia; SC—Sertoli cells; SCI—spermatocytes of the 1st order; SCII—spermatocytes of the 2nd order; SD—spermatids; M—myoid cells; C—capillary; IT—interstitial tissue with endocrinocytes.

The micrographs obtained show cells at all stages of spermatogenesis: from basally developed spermatogonia to mature spermatozoa located in the lumen of the tubule. Sertoli cells were present in all sections, which confirms the preservation of the hematotesticular barrier and trophic support of gametes. In the samples, these cells had an elongated shape, an oval light nucleus and extended from the basement membrane to the lumen of the tubule (Figure 3).

### 3.2. Morpho-Histology and Morphometry of Testes

In cross-sections on selected fields measuring 100 × 100 µm, the main types of spermatogenic epithelial cells were counted on 10 random sections of each testis (Table 3). For each indicator, the average values (M ± SE) were calculated. The epithelium of the seminiferous tubules contained cells at all stages of spermatogenesis: spermatogonia (13.9 ± 0.9 cells), spermatocytes of the first order (20.3 ± 4.2 cells), spermatocytes of the second order (51.0 ± 13.9 cells), spermatids (65.7 ± 18.9 cells), as well as Sertoli cells (3.6 ± 1.2 cells) (Table 3). The interstitial tissue contained weakly expressed Leydig cells, which corresponds to the typical histological picture of the reproductively active period in roosters at the age of 32 weeks of life.

The average number of seminiferous tubules in the field of view at magnification × 100 was 52.8 ± 9.0 (Table 3). The results of measuring the diameter of the cross sections of the seminiferous tubules showed values from 212.4 µm to 444.4 µm (MEAN ± SE 305.4 ± 8.7 µm). The variability of the diameter of the seminiferous tubules, estimated by the coefficient of variation (CV), was in the range of 17.9–20.4%, which indicates a moderate individual heterogeneity of morphometric parameters. The minimum value of the cross-sectional area of the seminiferous tubules was 44,638.6 µm^2^ (preparation from male No. 9), and the maximum was 115,027.6 µm^2^ (preparation from male No. 7); thus, the individual variability of the indicator (CV) was 40.8%.

### 3.3. Correlation Analysis Between Histological Parameters and Ejaculate Quality

**Figure 4:**
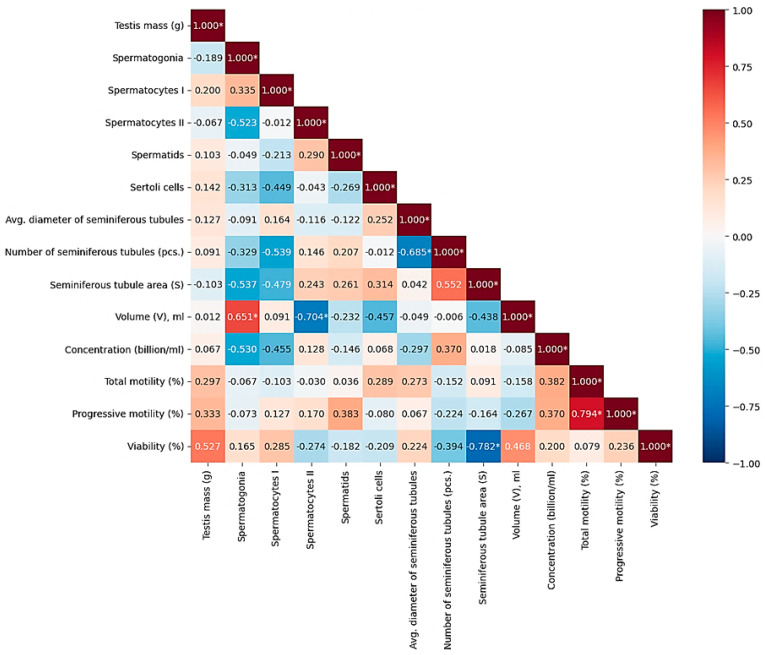
Correlations between morphometric and physiological parameters of rooster testicles and macro- and microscopic indicators of semen quality. Color scale: red—positive correlation, blue—negative, white—lack of correlation. Note: * significant correlations (*p* < 0.05).

As a result of the Spearman correlation analysis, several significant relationships were revealed between the morphometric parameters of the testicles and the indicators of fresh ejaculate in roosters (at *p* < 0.05) (Table 4). At n = 10 and the significance level *p* < 0.05, the critical value of the Spearman coefficient was |*p*| = 0.648; the relationships with |*p*| ≤ 0.648 were interpreted as statistically significant. Thus, a correlation was considered statistically significant if its absolute value was ≤0.05. To visualize the strength of correlations, a thermal correlation matrix with a rank scale was constructed (Figure 4).

According to the results of the evaluation of histological samples of rooster testicles by the composition of the spermatogenic epithelium, a positive correlation (r = 0.335) was noted between the number of spermatogonia and spermatocytes of the first order, which relate to the period of germ cell growth during spermatogenesis. Evaluating the quantitative indicators of the number of spermatocytes of the second order and spermatids, which belong to the next stage of spermatogenesis—the transition to the haploid state and the maturation period—a moderate positive correlation was also noted (r = 0.290). The connections were not reliable, but they were logical and predictable. There was no relationship between testicle weight (g) and sperm concentration (billion/mL) and/or ejaculate volume (ml), while the indicator of sperm concentration had a positive relationship with the indicator of the number of seminiferous tubules of the testis (r = 0.370, *p* < 0.05). The strongest positive reliable (*p* < 0.05) correlations were found between the number of mature spermatids in the spermatogenic epithelium of the seminiferous tubules and the indicators of sperm motility: the correlation coefficient with progressive motility (PM) was +0.794; between the number of spermatids and sperm viability (r = +0.761). These results indicate that the degree of completion of spermiogenesis, reflected by the number of spermatids, directly determines the quality of sperm. Significant positive associations were also found for other histological parameters. Thus, the number of spermatogonies positively correlated with the volume of ejaculate (r = +0.651), which may indicate that the activity of stem cell proliferation affects the productivity of the seminiferous tubule. In addition, the number of Sertoli cells demonstrated a moderately high and statistically significant relationship with sperm viability (r = +0.678), which emphasizes their role in providing trophic support and ensuring the integrity of the hematotesticular barrier. The revealed high negative correlation between the volume of ejaculate and the number of spermatocytes of the second order (*p* = −0.704) may indicate a shift in the balance of spermatogenesis with an increase in semen volume. A strong negative relationship was also found between the total cross-sectional area of the seminiferous tubules and sperm viability (*p* = −0.782). The negative correlation between the number of seminiferous tubules and their average diameter (*p* = −0.685) probably reflects a compensatory mechanism: as the number of tubules increases, their individual size decreases, and vice versa.

### 3.4. Analysis of TGFB2 and DMRT1 Gene Expression

**Figure 5:**
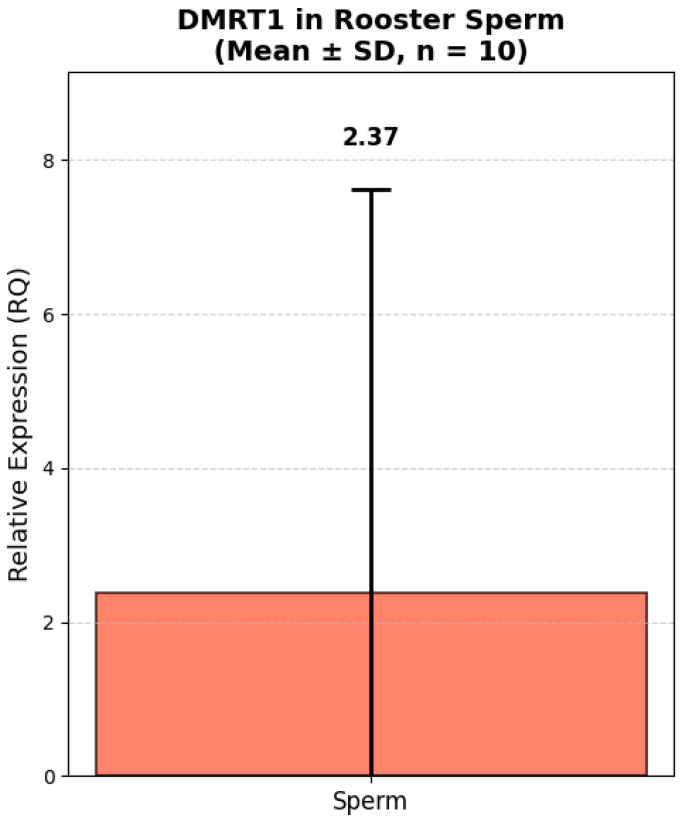
Relative expression of *DMRT1* in rooster spermatozoa (Mean ± SD, n = 10). Note: Relative expression was calculated using the ΔΔCt method with *GAPDH* as a reference gene. *GAPDH* expression was measured in kidney tissue due to lack of validated housekeeping genes for sperm in this study. All values are presented as mean ± standard deviation (SD) from 10 roosters.

**Figure 6:**
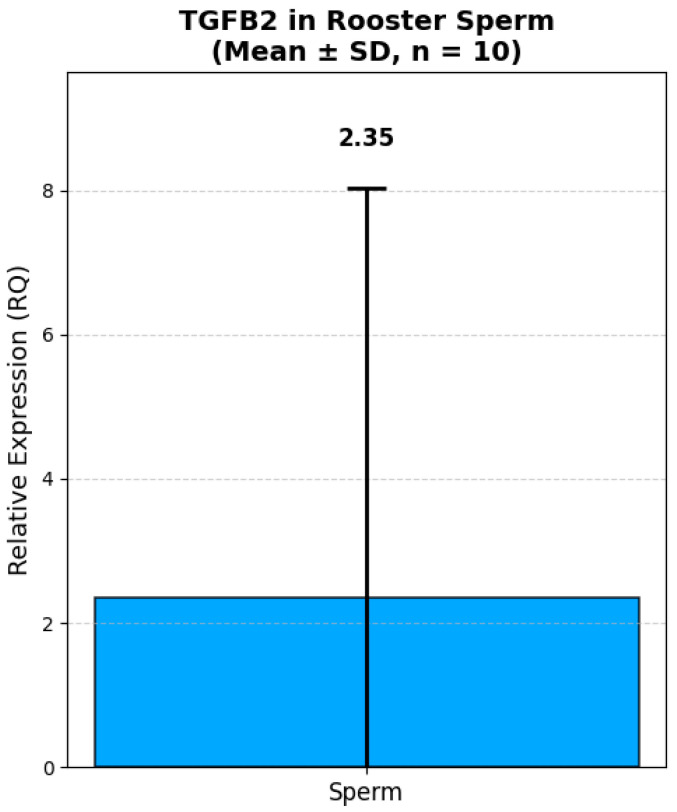
Relative expression of *TGFB2* in rooster spermatozoa (Mean ± SD, n = 10). Note: Relative expression was calculated using the ΔΔCt method with *GAPDH* as a reference gene. *GAPDH* expression was measured in kidney tissue due to lack of validated housekeeping genes for sperm in this study. All values are presented as mean ± standard deviation (SD) from 10 roosters.

Analysis of the relative expression of the *TGFB2* and *DMRT1* genes in fresh semen of Russian Snow White roosters revealed significant interindividual variability both in terms of the absolute level of transcripts and the expression ratio between the genes (Figure 5 and Figure 6). Thus, the expression level of the *TGFB2* gene ranged from 0.12 to 18.49 (in relative units, RQ). The majority of roosters (8 out of 10) had low or moderate expression of *TGFB2* (RQ < 1.0). Only one male, No. 2, had a sharply increased expression level of 18.49 RQ, which exceeded the group average by more than 10 times. The average expression value of the *TGFB2* gene was 2.59 ± 1.78 RQ. At the same time, the expression of the *DMRT1* gene also showed high variability: from 0.006 to 17.20 RQ. In 4 roosters, the expression level of *DMRT1* was extremely low (RQ < 0.1), while in male No. 2, it reached a maximum value of 17.20 RQ. The average *DMRT1* expression level in the sample was 2.91 ± 1.55 RQ. Interestingly, most roosters with low expression of *TGFB2* (RQ < 0.5) also had low or moderate expression of *DMRT1* (RQ < 1.0). The exception was sample No. 9, in which the expression of *TGFB2* was minimal (0.12 RQ), while the expression of *DMRT1* reached 2.21 RQ, one of the highest values in the sample.

The data obtained demonstrate that both genes are characterized by comparable levels of relative expression in the fresh semen. The proximity of the average values indicates a possible synchronized expression of these genes, which is consistent with their functional relationship in the regulation of spermatogenesis. High values of the standard error (SE > 1.6) reflect significant variability in expression levels between individuals, which may be due to individual differences in the physiological state and reproductive status of roosters.

The analysis of correlations between the relative expression of the *TGFB1* and *DMRT1* genes in fresh semen and the morphological/functional parameters of semen in 10 roosters revealed a number of statistically significant and biologically important relationships. The most pronounced and significant was the negative correlation between the level of *DMRT1* gene expression and sperm quality. It was found that increased *DMRT1* expression is significantly associated with a decrease in sperm motility and viability:

with progressive motility (r = −0.612, *p* < 0.05),

with total motility (r = −0.552, *p* < 0.05),

with viability (r = −0.552, *p* < 0.05).

**Figure 7:**
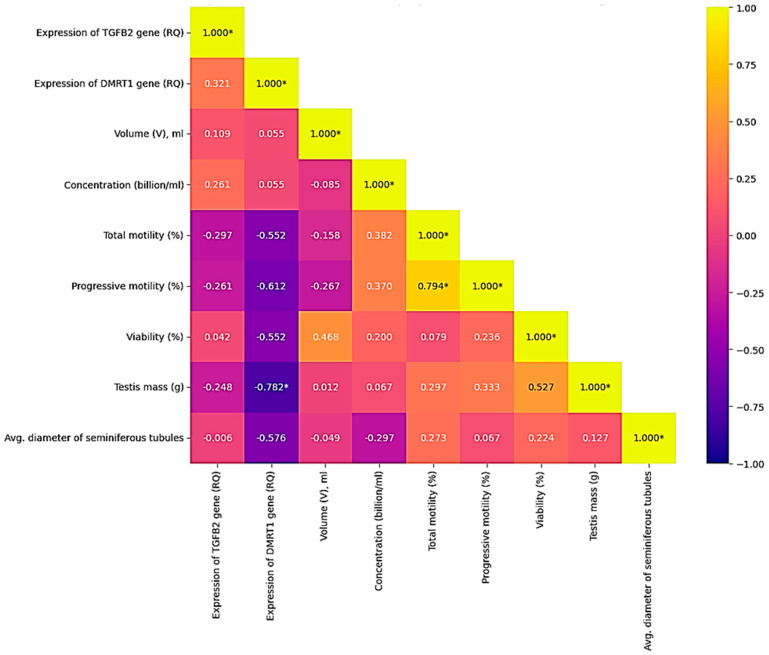
Correlations between the quality of fresh rooster semen and the expression levels of the *TGFB2* and *DMRT1* genes (*p* < 0.05). Color scale: yellow—positive correlation; blue—negative; blue—lack of correlation. Note: * significant correlations (*p* < 0.05).

In addition, the expression level of the *DMRT1* gene showed a strong negative correlation with testicle weight (r = −0.782, *p* < 0.05), which indicates that in roosters with larger testicles, the expression level of this gene in semen is significantly lower. At the same time, the expression of the *TGFB1* gene did not show statistically significant correlations with any of the studied parameters of sperm quality (motility, viability, concentration) or histological characteristics of the testicles (tubule diameter, number of cells, etc.). The only observed relationship is a weak positive correlation with *DMRT1* expression (r = 0.321), which also did not reach the level of statistical significance (Figure 7).

## 4. Discussion

Despite the homogeneous conditions of detention and the identical mode of semen sampling, significant variability in the morphometric parameters of the testicles was observed in our study—their weight ranged from 7.16 g to 25.47 g, which can be explained by individual differences in the level of circulating gonadotropins and androgens in individuals. According to Behnamifar et al. (2025), testosterone levels in the blood serum of roosters affect the morphology of the testicles and the quality of sperm production, including sperm concentration and viability [36]. Also, according to Lengyel et al. (2024), a violation of androgen signaling leads to impaired fertility even while maintaining the morphological integrity of the generative tissue, which confirms the importance of hormonal regulation in spermatogenesis [37]. In addition, our study demonstrated a significant variability (CV) in the size of the diameter of the cross–section of the seminiferous tubules by 20.4% (min diameter of the tubules is 212.7 µm; max is 444.4 µm), which is consistent with the data of other authors who studied the morphometry of reproductive organs in male poultry. For example, in a study by Mfoundou et al. (2022) in broiler chickens, the diameter of the seminiferous tubules before puberty at the age of 4 months was 110–130 µm [9]. Similar results were obtained in another study aimed at studying adaptive changes in the testicles of broilers aged 20–28 days of life (Islam et al., 2021 [14]). It was found that the diameter of the seminiferous tubules of the studied individuals was 135–145 µm, depending on the age of the chickens [14]. In a study by Razi M. Kugler P. (2010), the diameter of the seminiferous tubules of sexually mature roosters with highly active spermatogenic epithelium was 162 µm [38]. This observation is consistent with the well-known morphological principle, according to which an increase in the diameter of the seminiferous tubule is accompanied not only by an increase in its cross-sectional area, but also by the laying of more layers of spermatogenic epithelium [39]. Olawuyi et al. (2019) described that it is this morphometric dependence that allows the organ to adapt to the functional loads associated with spermatogenesis [39]. One of the key results was the detection of a strong negative correlation between the total cross-sectional area of the seminiferous tubules and sperm viability (*p* = −0.782). At first glance, this contradicts expectations, since an increase in the area of the epithelium is often associated with increased productivity. However, such an observation may reflect the functional heterogeneity of the tubules: in the presence of a large number of large tubules, microcirculation may be impaired, hormonal regulation may be unbalanced, or the effectiveness of supporting Sertoli cells may decrease. Similar results have been obtained in other studies conducted on mammals. For example, in a study by Crean et al. (2023), histological analysis of mouse testicles revealed that the average cross-sectional area of the seminiferous tubules negatively correlates with their number (r = −0.39, *p* = 0.002) [40]. The authors interpreted this as a compensatory mechanism in which a sufficient number of sperm can be produced either by increasing the diameter of the tubules (with a correspondingly smaller number on the cross-section) or by increasing the number of tubules of a smaller diameter [41]. A similar compensatory mechanism is also observed in the negative relationship between the number of seminiferous tubules and their average diameter (*p* = −0.685, *p* < 0.05). It is known that with a fixed volume of testicular tissue, there is a balance between the number of seminiferous tubules and their diameter, due to the need for optimal packaging and distribution of cells inside the gonads [42,43]. This allows the testicle to effectively use space to maximize the area of the spermatogenous epithelium while limiting the total volume of the parenchyma. Compensatory mechanisms in spermatogenesis can manifest themselves in changes in the speed of passage of germ cells through various stages of development. Thus, increased proliferation of spermatogonia in birds with a large volume of ejaculate may be accompanied by accelerated passage of second-order spermatocytes through the stages of meiosis II and early spermatidiation, which explains the negative correlation between the volume of ejaculate and the number of second-order spermatocytes [44]. At the same time, the revealed positive correlation between the volume of ejaculate and the number of spermatogonies (r = 0.651) may indicate that males with a large semen volume have more intense initial stages of spermatogenesis, in particular, the proliferation of spermatogonies. A study by O’Donnell et al., 2017, described that this stage is a hormone-modulated process that can be activated when the demand for sperm production increases [41]. This is consistent with the concept of “demand-driven spermatogenesis”, according to which its intensity adapts to the physiological needs of the body. With increased activity of the accessory glands, sperm production increases, thereby intensifying the proliferation of spermatogonia. It is noteworthy that in our study, such a significant indicator as the concentration of spermatozoa did not have significant correlations with most morphometric parameters of the testicles. Similar results were described in a study by Sun et al. (2019), which also revealed complex, nonlinear relationships between testicle morphometry and sperm quality parameters in Beijing-You roosters aged 40–44 weeks [22]. It was found that males with low sperm motility had low semen volume and sperm viability; however, correlations between the morphometric parameters of the testicles and the concentration of ejaculate were either weak or absent. The authors explained this by the fact that semen quality depends not so much on the structural parameters of the generative tissue of the reproductive organs as on the intensity of germ cell apoptosis, the functional state of the testicle appendage, and other factors [22]. In addition, Liu et al. (2023) demonstrated in their study that the sperm DNA fragmentation index (DFI) negatively correlated with the viability, concentration, and progressive motility of germ cells, but had no relationship with ejaculate volume [44]. This confirms that the various parameters of sperm production are regulated independently of each other and do not always correlate with the morphometric parameters of the testicles.

The development of male reproductive organs, starting from the period of postnatal development to puberty, is characterized by intensive proliferation of Sertoli cells and their subsequent differentiation [45]. The low number of Sertoli cells found in our study (3.6 ± 1.2 in the ×400 field of view) with a sufficiently high number of developing germ cells fully corresponds to the data from the scientific literature, according to which one Sertoli cell is capable of supporting approximately 30–50 gametes [38,45,46]. The low number of Sertoli cells in roosters in relation to basally oriented germ cells, compared with other species, is explained by their high cytochemical activity of diaphorase, lactate dehydrogenase, succinate dehydrogenase, cytochrome oxidase, which are metabolically more independent of Sertoli cells [39]. In a meta-analysis conducted by Rebourcet et al. (2017), they found that there is a strong correlation between the number of Sertoli cells formed during the active reproductive period and the total number of gametes in adulthood (r = 0.800; *p* < 0.001), which once again confirms the importance of these cells in spermatogenesis [47]. However, a study by Wilson et al. (2018) revealed that in roosters that have reached puberty (~29 weeks), there is a gradual decrease in the concentration of Sertoli cells in the spermatogenic epithelium, which is associated with the onset of active spermatogenesis and the completion of the formation of reproductive organs [20]. The negative correlation observed in our study between the number of Sertoli cells and first-order spermatocytes (*p* = −0.449) did not reach the threshold of statistical significance; however, it may reflect the phase dynamics of the spermatogenic cycle: in the areas of the seminiferous tubules dominated by the meiotic stage, the relative density of Sertoli cells decreases due to an increase in the number of germinal cells. Alternatively, this may be due to a methodological limitation—the partial overlap of Sertoli cell nuclei with a high density of spermatocytes in the visual field [48].

Of particular interest in our study is the analysis of the relationship between the expression level of key genes involved in the regulation of spermatogenesis and indicators of both the histological structure of the testicles and the functional quality of fresh semen. The most significant results relate to the negative relationship between *DMRT1* gene expression and key indicators of reproductive function, which requires special attention and interpretation. For the gene *DMRT1*, our results showed a mean relative expression (RQ) of 2.38 ± 5.44 in spermatozoa. Although this value is relatively low compared to other tissues, it is biologically significant given that *DMRT1* is a master regulator of male sexual development and is known to be highly expressed in testicular tissue. Indeed, Bgee data (https://www.bgee.org/, accessed on 17 November 2020) confirm that *DMRT1* exhibits its highest expression rate (91.86) in testicular tissue, followed by spermatogenic epithelial cells of the testes—spermatocytes (87.47) and spermatids (85.20). The presence of detectable *DMRT1* transcripts in mature spermatozoa suggests that residual mRNA from earlier stages of spermatogenesis may persist, potentially playing a role in posttesticular maturation and capacitation. Also, published data from Bgee on extremely low or undetectable levels of *DMRT1* expression in somatic tissues such as the kidney (25.83) and heart (17.92) indicate its modest role in the regulation of these organs.

Firstly, the expression level of the *DMRT1* gene significantly negatively correlates with testicle weight (r = −0.782, *p* < 0.05). This means that in roosters with larger testicles, the activity of the *DMRT1* gene in spermatozoa is reduced. This seemingly paradoxical result may be due to the fact that *DMRT1* is a key regulator of the early stages of spermatogenesis and sex determination in birds [49]. In mature testicles with high productivity, where spermatogenesis proceeds intensively, the main expression of *DMRT1* is localized in testicle tissue cells (spermatogonia, Sertoli cells), whereas in mature spermatozoa, where the residual transcriptome is active, the level of *DMRT1* may be reduced. Thus, the obtained value may reflect not the expression of a gene in semen, but the productive activity of the tissue, where the regulatory function of *DMRT1* has already been implemented at previous stages of differentiation. Secondly, the level of *DMRT1* gene expression in spermatozoa is negatively related to the functional quality of sperm: negative correlations were found with total motility (r = −0.552), progressive motility (r = −0.612) and viability (r = −0.552). This suggests that an increased *DMRT1* level in mature spermatozoa may be a marker of untimely or impaired spermatogenesis. Normally, as spermatids transform into spermatozoa, histones are globally replaced by protamines, and most of the nuclear RNA is displaced [50]. Maintaining a high level of the *DMRT1* gene transcript, which is active in the early stages, may indicate the incompleteness of this process, which is likely to negatively affect the morphofunctional maturity and viability of gametes [24]. In addition, the expression level of the *DMRT1* gene in spermatozoa and the average diameter of the cross-section of the seminiferous tubules had a negative correlation (*p* = −0.576), which, although it does not reach the threshold of statistical significance, is consistent with the well-known role of *DMRT1* as a regulator of the early stages of germinal tissue differentiation. A decrease in the expression of this gene in adulthood probably reflects the transition of the testicles from the phase of active differentiation to the phase of functional support of spermatogenesis, accompanied by an increase in the morphometric parameters of the tubules.

Unlike *DMRT1*, the expression of the *TGFB2* gene showed no statistically significant correlations with either histological or semen quality indicators. The gene *TGFB2* displayed a similar mean RQ (2.35 ± 5.33) in sperm, but its expression pattern across tissues is markedly different. Bgee data show that *TGFB2* is expressed at high levels in many tissues, such as skeletal muscle (74,76), brain (63,20), lung (54,57), and at moderate levels in testicular (44,20) and kidney (30,16) tissues. This is logical, since this gene is involved in the TGF-β growth factor signaling pathway. The detection of *TGFB2* mRNA in spermatozoa, while not unexpected, raises intriguing questions about its potential role in sperm function or fertilization. Given that TGF-β signaling has been implicated in sperm capacitation and embryo-maternal communication, even low-level expression in sperm could have functional relevance. This is consistent with the well-known role of the TGF-β signaling pathway as a regulator of proliferation, differentiation, and apoptosis of germ and somatic cells directly in the spermatogenic epithelium of the seminiferous tubules, rather than in mature spermatozoa [6,51]. Our study, which focuses on the expression of these genes in semen, cannot reflect the activity of this pathway at the testicle tissue level, which probably explains the lack of identified connections. Interestingly, no direct correlation was found between the *TGFB2* and *DMRT1* genes themselves (r = 0.321), which indicates an independent regulation of their expression in mature spermatozoa. This result highlights the need to consider each of these genes as a separate molecular marker. The data obtained complement the known information that the quality of sperm is determined not only by quantitative characteristics (volume, concentration), but also by the molecular maturity of germ cells. Our study demonstrates that the level of *DMRT1* expression in fresh semen can serve as a prognostic marker of the functional quality of spermatozoa: its low level is associated with high mobility and viability, while increased expression indicates potential disorders in the process of spermiogenesis. Further research should be aimed at studying the mechanisms of compensatory regulation at the molecular level, including analysis of the expression of genes regulating apoptosis and germ cell proliferation, as well as assessment of circulating hormone levels in birds and their relationship to various morphometric parameters of the testicles.

## 5. Conclusions

As a result of a comprehensive study, key morphometric, cellular, and molecular parameters that affect semen quality in roosters (*Gallus gallus domesticus*) were identified. It has been shown that the maturity of the spermatogenic epithelium and the structural organization of the seminiferous tubules are closely related to the parameters of sperm motility and viability. It was also found that high expression of the *DMRT1* gene in rooster semen may be a marker of immaturity of the spermatogenic process, while the *TGFB2* gene performs background regulatory functions (the regulation of spermatogenesis and testicle development) at the molecular level under non-selective conditions. The data obtained indicate the expediency of integrating morphometric, cellular and molecular analysis for an objective assessment of rooster reproductive function.

## Figures and Tables

**Table 1:** The nucleotide sequence of primers for RT-PCR in real time.

Name of the Gene	Sequence of Oligonucleotides
*TGFB2*	Forward: GAAGCTTCTGCCTCTCCGTG
	Reverse: GTCACGCTGTTTCTGGGGTA
*DMRT1*	Forward: CACACAGATACTGGCCTCGG
	Revers: TAAGTCGAGGCACTCAACGC
*GAPDH*	Forward: CGCCATCACTATCTTCCAGG
	Reverse: CCTCTGTCATCTCTCCACAGC

**Table 2:** Indicators of fresh semen in roosters aged 28–30 weeks.

No.	Testicles Weight, g, ±SE *	Ejaculate Volume, mL, ±SE	Sperm Concentration, Billion/mL, ±SE	Total Motility of Spermatozoa, %, ±SE	Progressive Motility, %, ±SE	Sperm Viability, %, ±SE
1	16.88	0.78 ± 0.09	2.37 ± 0.31	92.1 ± 1.2	79.4 ± 10.2	85.1 ± 11.1
2	13.21	0.41 ± 0.100	3.26 ± 1.20	72.8 ± 13.1	55.7 ± 15.7	74.4 ± 0.1
3	15.47	0.39 ± 0.03	3.02 ± 0.96	90.6 ± 3.6	81.4 ± 9.7	70.5 ± 3.9
4	8.62	0.38 ± 0.04	1.73 ± 0.33	65.0 ± 16.7	54.7 ± 8.8	71.7 ± 2.9
5	25.47	0.79 ± 0.09	1.36 ± 0.70	81.8 ± 3.5	67.1 ± 4.9	77.3 ± 7.1
6	7.16	0.48 ± 0.01	1.51 ± 0.55	82.4 ± 3.8	66.7 ± 7.0	65.4 ± 8.9
7	17.09	0.38 ± 0.04	1.70 ± 0.36	88.8 ± 3.3	73.1 ± 6.5	80.2 ± 6.1
8	16.26	0.20 ± 0.09	2.19 ± 0.13	76.2 ± 18.5	50.7 ± 13.5	69.7 ± 4.9
9	10.64	0.63 ± 0.04	1.08 ± 0.98	73.1 ± 16.3	47.7 ± 18.1	70.1 ± 3.9
10	10.62	0.79 ± 0.09	1.79 ± 0.27	87.4 ± 2.7	77.3 ± 2.5	78.8 ± 4.1
M ± SE	14.08 ± 5.98	0.50 ± 0.20	2.06 ± 0.72	80.8 ± 2.7	65.4 ± 3.9	74.3 ± 1.9

Note: * SE—standard error.

**Table 3:** Cellular composition of the spermatogenic epithelium of the seminiferous tubules of roosters (*Gallus gallus domesticus*) at the age of 32 weeks of life.

No.	Weight of the Right Testicle, g	Number of the Seminiferous Tubules *	Diameter of the Cross Section of the Seminiferous Tubule, µm	The Area of the Seminiferous Tubules in the Field of View, * (µm^2^) ±SE	Number of Spermatogonia, ** ±SE	Number of Spermatocytes of the 1st Order, ** ±SE	Number of Spermatocytes of the 2nd Order, ** ±SE	Number of Spermatids, ** ±SE	Number of Sertoli Cells, ** ±SE
1	16.88	55	251.2 ± 3.9	50,949.6 ± 850.8	15.0 ± 0.6	19.7 ± 3.5	42.0 ± 8.0	65.0 ± 14.3	3.3 ± 0.7
2	13.21	86	243.9 ± 13.2	47,252.8 ± 5258.9	13.0 ± 1.0	16.0 ± 1.5	43.7 ± 4.3	49.0 ± 6.6	4.7 ± 0.3
3	15.47	68	321.1 ± 18.0	81,959.8 ± 9710.0	12.3 ± 3.8	19.3 ± 3.7	70.7 ± 15.8	98.0 ± 10.7	2.7 ± 0.7
4	8.62	39	309.9 ± 24.0	77,215.6 ± 12,000.6	13.7 ± 1.9	20.0 ± 1.2	56.3 ± 17.2	102.0 ± 13.7	3.0 ± 0.6
5	25.47	56	340.2 ± 15.5	91,582.7 ± 7737.9	14.0 ± 0.6	20.3 ± 2.4	40.7 ± 15.3	69.3 ± 12.4	3.3 ± 0.9
6	7.16	43	353.8 ± 39.1	103,075.4 ± 20871.9	14.3 ± 1.8	18.0 ± 5.0	43.3 ± 8.7	54.7 ± 5.8	5.0 ± 0.6
7	17.09	28	381.7 ± 14.6	115,027.6 ± 8850.4	13.3 ± 1.2	26.7 ± 2.4	61.3 ± 7.7	49.7 ± 2.9	4.7 ± 0.3
8	16.26	60	299.5 ± 13.5	70,994.3 ± 6475.9	13.3 ± 1.7	25.7 ± 2.2	44.7 ± 19.3	57.3 ± 1.5	4.7 ± 1.2
9	10.64	61	237.2 ± 12.3	44,638.6 ± 4712.2	15.0 ± 2.1	26.0 ± 2.0	56.3 ± 1.8	65.0 ± 1.0	2.3 ± 0.9
10	10.62	34	325.9 ± 27.6	85,780.5 ± 15,073.5	14.6 ± 1.7	28.0 ± 3.6	41.6 ± 8.2	46.6 ± 5.0	1.6 ± 0.3
M ± SE	14.08 ± 5.98	52.8 ± 6.1	305.4 ± 8.7	76,847.4 ± 4429.7	13.9 ± 0.9	21.9 ± 1.1	51.0 ± 10.8	65.7 ± 18.9	3.6 ± 1.2

Note: * in the field of view at ×100 magnification; ** in the field of view at ×400 magnification; SE—standard error.

**Table 4:** Significant correlations between histomorphological parameters, gene expression, and ejaculate quality in roosters (n = 10, *p* ≤ 0.05).

A Couple of Variables (↔)	Correlation Coefficient (r)	Interpreting the Strength of the Bond
Ejaculate volume (V, mL) ↔ Spermatogonia	+0.651	Moderate positive
Ejaculate volume (V, mL) ↔ Spermatocytes of the second order	–0.704	High negative
Area of seminiferous tubules (S) ↔ Viability (%)	–0.782	High negative
Number of seminiferous tubules (pcs.) ↔ Average diameter of the cross section	–0.685	High negative
Total motility (%) ↔ Progressive motility (%)	+0.794	High positive
Testicle weight (g) ↔ *DMRT1* (RQ) gene expression	–0.782	High negative

## Data Availability

The original contributions presented in this study are included in the article. Further inquiries can be directed to the corresponding author.
